# Effect of Carbon Dioxide Loading on Removal of Heat Stable Salts from Amine Solvent by Electrodialysis

**DOI:** 10.3390/membranes9110152

**Published:** 2019-11-13

**Authors:** Evgeniia Grushevenko, Stepan Bazhenov, Vladimir Vasilevsky, Eduard Novitsky, Maxim Shalygin, Alexey Volkov

**Affiliations:** A.V. Topchiev Institute of Petrochemical Synthesis RAS, Russian Academy of Sciences, Moscow 119071, Russia; evgrushevenko@ips.ac.ru (E.G.); sbazhenov@ips.ac.ru (S.B.); vasilevskii@ips.ac.ru (V.V.); ednov@ips.ac.ru (E.N.); MShalygin@ips.ac.ru (M.S.)

**Keywords:** heat stable salts, monoethanolamine, electrodialysis, reclaiming, carbon dioxide

## Abstract

Heat stable salts (HSS) formed and continuously accumulated in the amine-based solvents due to solvent degradation and impurities in the feed gas can dramatically change the efficiency of the amine scrubbing process. HSS can be removed by using different methods including membrane separation such as electrodialysis (ED). In this work, we studied the effect of CO_2_ loading of the lean 30 wt % monoethanolamine (MEA) solution on the efficiency of HSS removal and MEA loss. In the model MEA solution containing HSS on the level of 48 meq/L, the carbon dioxide concentration was varied from 0.2 down to 0 mole (CO_2_)/mole (MEA). The reclaiming of model MEA solution was carried out by lab-scale two-stage ED unit when the concentrate stream after the first stage was additionally treated using ED (second stage) that allowed reducing MEA loss. It was shown that the decrease of carbon dioxide content from 0.2 down to 0 mole (CO_2_)/mole (MEA) resulted in a substantial reduction of both parameters—the MEA loss and the specific power consumption with respect to extracted HSS (from 140 down 37 kJ per 1 g of recovered HSS anions). This can be explained by the drop in the total concentration of ions formed by the interaction of MEA solution with carbon dioxide. However, the change of CO_2_ loading is associated with additional power consumption towards further solvent regeneration in the column. Based on the preliminary estimations of power consumption required for additional CO_2_ stripping with the respect to the power consumption of ED stage, it seems that lean solvent CO_2_ loading of 0.1 mole/mole provides an optimum for the power input at 25.9 MJ/kg(solvent).

## 1. Introduction

**Figure 1:**
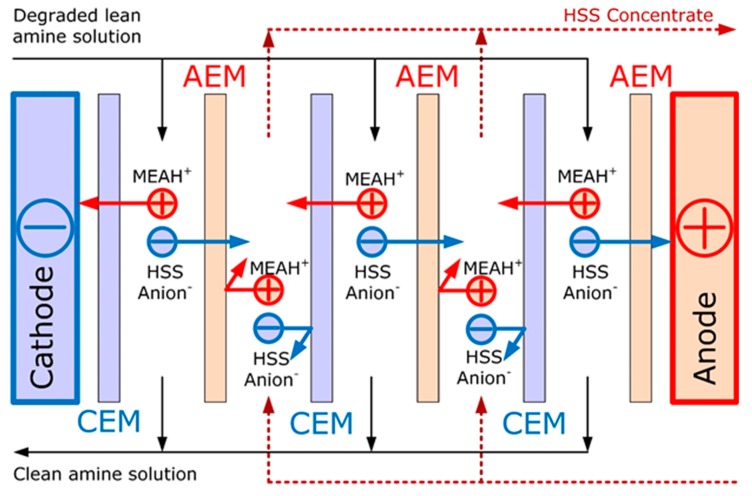
The principle of heat stable salts (HSS) removal from monoethanolamine solvent with electrodialysis (CEM—cation-exchange membrane, AEM—anion-exchange membrane).

Amine scrubbing is the most widespread technology for carbon dioxide capture from the technological and flue gases [1,2]. However, one of the major drawbacks of this technology is the degradation of amine solvent due to the high temperature of regeneration (100–130 °C) and the presence of oxygen (e.g., in case of flue gas treatment). Furthermore, alkanol ammonium cation interacts with anions of organic (products of amine destruction) and inorganic (SO_x_, NO_x_ presented in the feed gas, impurities in make-up water) acids by forming of heat stable salts (HSS) [3,4]. HSS are stable and do not decompose at the conditions typical for solvent regeneration. The accumulation of HSS in the absorption system leads to such operational problems as the decrease of the absorbent CO_2_ capacity and change in its physicochemical properties, increase in the corrosiveness and consequent clogging and equipment erosion [5]. HSS can be removed from the amine solvent by means of distillation (process by Gazprom VNIIGAZ [6] and CCR Technologies Ltd. [7]), ion exchange (process MPR CCAR™ and AmiPur^®^–CCS by MPR Servises, Inc and Eco-Tec, correspondingly [8]) or electrodialysis [9,10,11,12,13,14,15,16]. The distillation process enables to remove all major impurities, but it is an energy-intensive process since HSS together with other non-volatile products of degradation (e.g., resins) are accumulated in the bottom residue, while the most of alkanolamine and water are evaporated. At the same time, the amine solvents can be reclaimed without phase change by ion-exchange [8,17,18,19] or electrodialysis (ED) [12,13,14,15,16]. Electrodialysis possesses several advantages typical for membrane processes: compactness, modularity, the flexibility of exploitation and easiness of upscaling [20,21]. The principle of HSS removal from monoethanolamine (MEA) solvent by electrodialysis is schematically shown on Figure 1. Studies are conducted on the research of the influence of ion exchange membrane surface modification on the intensity and selectivity of ion transport [22,23,24]. However, both approaches enable to remove the charged species including HSS components, and the additional treatment like sand filter and active carbon might be required for removal of the neutral degradation products.

**Figure 2:**
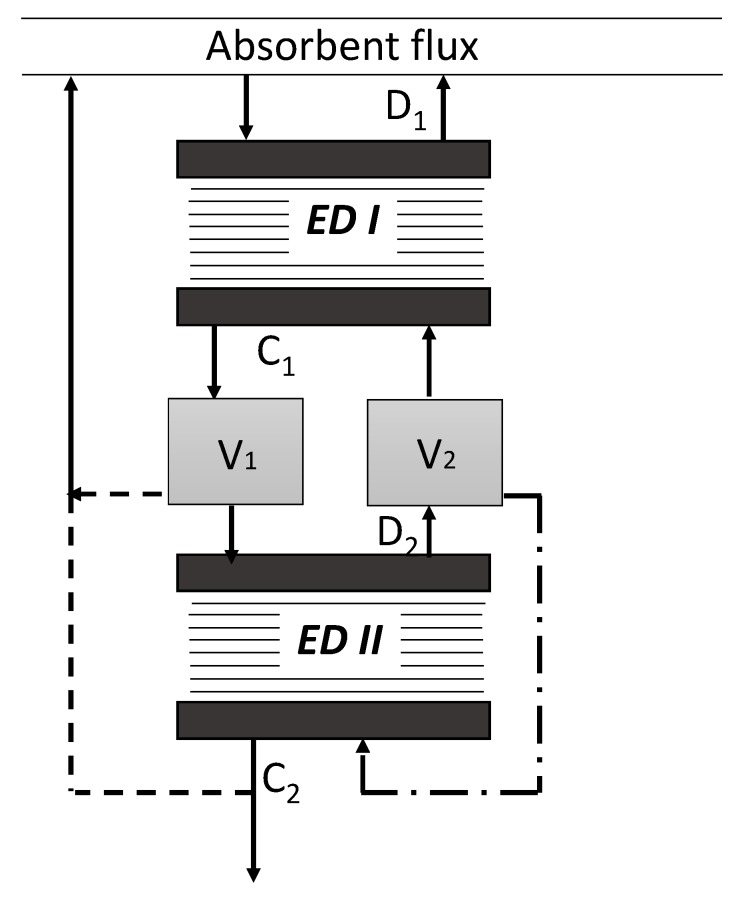
Flow scheme of two-stage electrodialysis (ED) system. D_1_—first stage dilute, C_1_—first stage concentrate, D_2_—second stage dilute, C_2_—second stage concentrate, V_1_ and V_1_—buffer tanks.

It should be pointed out that any reclaiming process is accompanied by the generation of the waste stream containing HSS components—bottom residue (distillation process), stream after reactivation of ion-exchange resin (ion-exchange process), concentrate stream (electrodialysis process). Most importantly, such waste is also associated with a certain loss of amine solvent. For instance, this aspect was considered within the works of Lim et al. [10,11], where the authors showed that permeation rates of MEA in electrodialysis were in the range of 0.054–0.82 g·m^−2^·s^−1^ with the respect to the type of membranes. The authors stated that most of amine was transferred through the membrane as free amine, which can be explained by its diffusion rather than by electromigration. Wang et al. [16] also found out the small decrease of N-methyldiethanolamine in the feed solution during the ED reclaiming due to concentrate-gradient osmosis and electro-osmosis mechanisms. However, the influence of CO_2_-loading of the solvent to be ED-treated was not studied in these works. Our previous results during the pilot campaign on ED reclaiming of lean solution of MEA revealed the MEA loss on the level of 33–109 mole to 1 mole of HSS removed with the respect to initial HSS content [12]. The follow-up study revealed that the MEA loss can be further reduced by additional ED reclaiming of the concentrate stream generated on the first stage [25]. At the same recovery rate of HSS components, two-stage ED process demonstrated MEA loss of 9.9 mol to 1 mol of HSS, which is twice lower as for one-stage ED process, 33 mole (MEA)/mole (HSS). Two-stage approach, which flow scheme is presented on Figure 2, further reduces the waste (concentrate) stream by half, which is 14% of the initial volume of amine. However, such improvements are associated with an increase of the required membrane area by 20% and the specific power consumption from 150 to 240 MJ/kg (HSS) comparing with one-stage ED reclaiming process.

In contrast to the natural gas sweetening, the CO_2_ loading in the lean amine stream of the post-combustion process is rather high, typically around 0.2 mole (CO_2_)/mole (amine). In the electrodialysis, all charged species contained in the solution are migrated with the different rates from the feed to the concentrate compartment. Thus, the effectiveness of HSS removal and specific power consumption would drop down with the increase of CO_2_ loading due to the higher ratio of amine molecules presented in the charged form (monoethanolammonium and carbamate). Since ED reclaiming is more feasible to apply for treatment of slip-stream rather than the whole stream of lean solvent, the further decrease of CO_2_ loading can be achieved by introduction of additional stripping column or membrane desorber operated at higher temperature and/or vacuum. Bearing this in mind, the goal of this work was to investigate the effect of CO_2_ loading on the removal of heat stable salts from amine-based solvent. In this study, monoethanolamine was selected as the most studied solvent considered for carbon dioxide capture, and the CO_2_ loading was varied from 0.2 down to 0 mole/mole.

## 2. Materials and Methods

### 2.1. Materials

For the preparation of model solution containing HSS and CO_2_, the following chemicals were used: monoethanolamine (Ekos-1, Moscow, Russia), carbon dioxide (MGPZ, Moscow, Russia), formic acid, glacial acetic acid, oxalic acid dehydrate, nitric acid, sulfuric acid (Khimmed Sintez, Moscow, Russia), distilled water. 30 wt % solution of MEA in water was prepared gravimetrically, then the corresponded amount of inorganic and organic acids were added; then carbon dioxide was introduced to achieve the required CO_2_-loading. The concentration of each individual HSS anions is listed in Table 1, and represents its content in 30 wt % MEA solution after 972 h of operation in the pilot post-combustion plant operated the bituminous coal-fired power plant [12].

### 2.2. Electrodialysis Reclaiming

**Figure 3:**
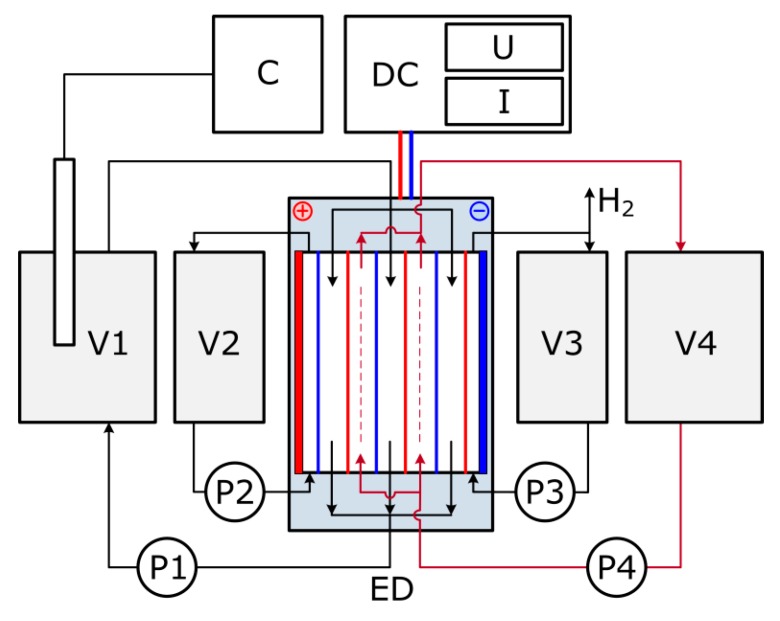
Lab-scale ED setup flow scheme: V1—dilute (feed) tank, V2—pre-cathode tank, V3—pre-anode tank, V4—concentrate tank, P1–4—pump, ED—electrodialyzer, DC—power supply, C—conductometer.

Electrodialysis was carried out in the controlled potential mode at 30 V voltage, the current mode was varied in the range 0.8–2.5 А (power supply Mastech HY5005E-2, Hong Kong, China) with the respect to MEA solution composition. The volume of the feed solution was 1 L. The initial concentrate stream was 30 wt % of MEA in water having the same CO_2_ loading as the feed to avoid amine diffusion between different compartments at the beginning of experiment due to concentration difference. The electrodialyzer with active surface area of about 20 dm^2^ was equipped with commercial cation-exchange membranes MK-40 and anion-exchange membranes MA-41 (ShchekinoAzot Ltd., Tula region, Russia) to obtain 10 desalting and 9 concentrating cells. Both electrodes are made of titanium covered by palladium. The rinse of electrode compartments was 30 wt % of MEA in water. The flow scheme of lab-scale ED stack is presented on Figure 3. On the first stage of ED treatment (ED I), 30 wt % water solution of MEA with an overall HSS anions concentration of 48 mmol-eq/l and varied CO_2_ loading, 0, 0.05, 0.1, 0.15 and 0.2 mole (СО_2_)/ mole (MEA), was used as the feed. The value of H for initial solutions was between 9 (0.2 mole (СО_2_)/ mole (MEA)) and 11 (0 mole (СО_2_)/ mole (MEA)). Once the overall HSS anions concentration of 89 mmol-eq/l in the concentrate stream was reached, the experiment was stopped, and then the feed solution was replaced by the fresh MEA solution. The experiment was replaced to collect the required volume of the concentrate solution, which was used as a feed for the second stage of ED treatment. In the case of zero CO_2_ loading of the model solution, the electrodialysis on the second stage was not carried out.

The specific energy consumption (*Qs*, kJ/g (HSS)) for the ED process was calculated based on experimentally obtained data according to the Equation (1):

(1) $$Qs=\frac{U\cdot I\cdot t}{m\left(HSS\right)}$$

where *U* (V) is the voltage applied to the electrodes, *I* (A) is average current for a period of time *t* (h) and *m*(HSS) (g) is HSS mass removed over a period of time *t* (h).

### 2.3. Analysis of Solvent Composition

During the experiment, the total HSS content was controlled by the comparison of solution conductivity (conductometer MultiLine P4, Weilheim, Germany) with the calibration curve of electroconductivity vs. HSS content. After the experiment, the concentration of individual ions was determined using the ionic chromatograph (“Akvilon Stayer-М“, chromatographic column Shodex ICSI-50 4E, eluent—3.2 mmole NaHCO_3_ and 0.1 mmole Na_2_CO_3_) equipped with the electromembrane suppressor EMCES 21 and conductometric detector CD-510. The error in determining the HSS concentration anions was not greater than 3%. The concentrations of MEA and CO_2_ were determined by titration of solution samples with 1 N HCl solution (Khimmed Sintez, Russia). The error in determining the MEA and CO_2_ concentration anions was not greater than 7 and 10%, respectively. The value of pH was determined using portable pH-meter Hanna HI 991003N. The first point of equivalence (pH = 7) allows to define the free protonated MEA unreacted with CO_2_, the second point of equivalence (pH = 4) corresponds to the reaction between HCl and MEA bound with СО_2_. Their sum was equal to the total MEA concentration; and CO_2_ loading as the concentration of bound carbon dioxide was determined from the difference between these two values.

### 2.4. Determination of Heat Consumption of Stripping Column by Aspen Plus^®^

The additional stripping column for the reduction of CO_2_ loading of the lean solution from 0.2 mole/mole down to required level was simulated by using Aspen Plus^®^ V.8.6 (ELECNRTL model). The goal of this study was to preliminary compare the electric power consumption of ED unit with heat consumption required for additional stripping. Other costs including CAPEX as well as column design optimization with the respect to resulted CO_2_ loading were out of scope of this work. Therefore, the mass transfer surface area was deliberately set higher than necessary to provide the equal conditions for all the cases. The fixed input parameters were the lean 30 wt % MEA and CO_2_ loading of 0.2 mole/mole, then the heat consumption of desorber reboiler required to achieve the target CO_2_ loading was determined.

## 3. Results and Discussion

### 3.1. Transport of HSS Anions

**Figure 4:**
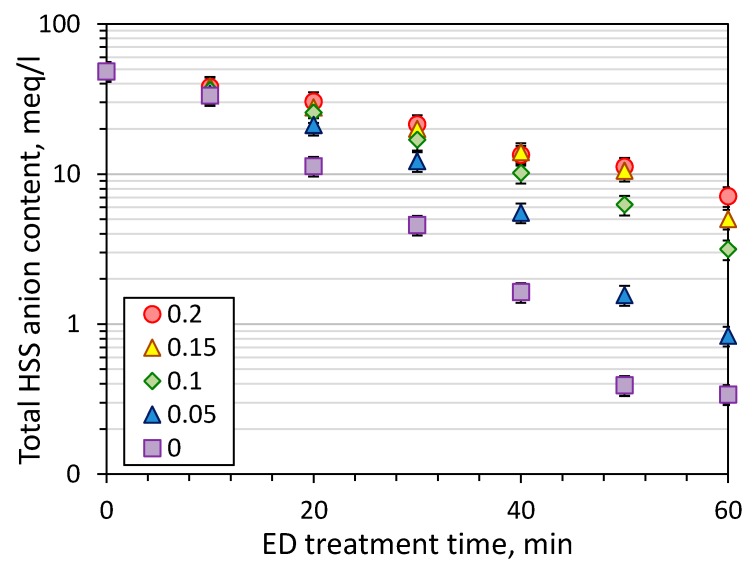
The change of total HSS anions concentration in the feed in time with the respect to initial CO_2_ loading (ED I).

Figure 4 shows the kinetics of HSS anions removal during the first stage of electrodialysis treatment of model MEA solution. The pronounced decline of HSS content in the feed was observed starting from the first 10 min of operation, and it can be seen that CO_2_ loading plays a major role in the effectiveness of ED process. For instance, 85% recovery of HSS anions from MEA can be achieved within about 25 min of ED treatment of amine solution without carbon dioxide and 60 min in the case of CO_2_ loading of 0.2 mole/mole. The HSS anions recovery after one hour of ED treatment was increased with the decrease of CO_2_ loading: 85 ± 4% for 0.2 mole/mole, 90 ± 2% for 0.15, 93 ± 2% for 0.1, 98 ± 1% for 0.05, 99 ± 1% for 0. Two conclusions can be made based on this data. Firstly, there is no need to decrease CO_2_ loading of lean solution lower than 0.05 mole/mole due to very close HSS recovery comparing with MEA solution without carbon dioxide—98 and 99%, respectively. Starting with CO_2_ loading of 0.05 mole/mole or greater there is a linear drop of the HSS recovery with an increase of carbon dioxide concentration due to the greater content of charged species in the solution and their competitive transport through the membrane.

**Figure 5:**
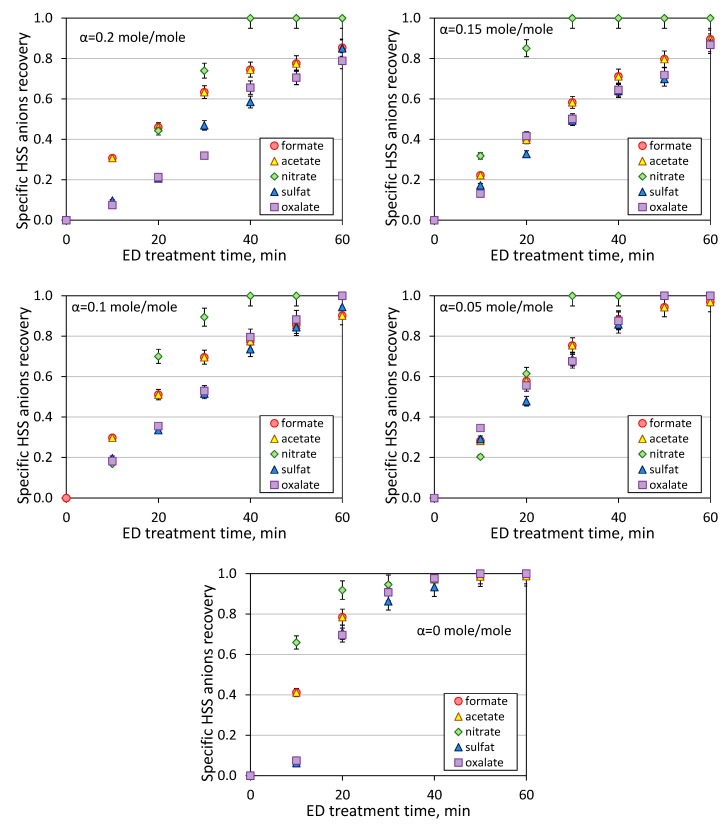
Kinetic of specific HSS anions recovery at different CO_2_-loading.

As mentioned earlier in the experimental part, both circuits of ED unit had the same composition (MEA content and CO_2_-loading) except the presence of HSS anions (see Table 1) in the feed compartment. Thus, the migration of different components through the membrane was mainly because of the electrodialysis process rather than the diffusion due to the concentration difference. Based on analysis of chemical composition and volume change of the feed, it was possible to determine the change of concentration of each individual HSS anion during the course of ED reclaiming (Figure 5). Among all HSS anions and regardless the CO_2_ loading, the fastest recovery was found for nitrate anions (inorganic, monobasic, strong acid; pKa = −1.64), which can be explained by its presence in the dissociated form in the solution and higher mobility within the membrane. There was an opposite situation for divalent acids, both inorganic and organic one, sulfate (pKa_1_ = −3, pKa_2_ = 1.9) and oxalate (pKa_1_ = 1.25, pKa_2_ = 4.27) ions demonstrated the lowest removal rate. Despite noticeable differences in acidity constants, these two HSS anions showed quite similar behavior. It is interesting to notice that the anions charge has a greater contribution in the mobility rather than dissociation degree of the acid molecules since formate (pKa = 3.75) and acetate (pKa = 4.76), anions of two weakest organic acids among studied ones, showed higher efficiency in the recovery over sulfate and oxalate [12]. Bearing this mind, it can be concluded that the anions charge effect shall play a pronounced role in the ions; for instance, the ions with higher charge possessed lower mobility due to greater retaining by the resin used in the ion-exchange membranes [26,27]. Additionally, the size of solvation shell would also impact the ions mobility within the membrane; for example, the solvation shell for nitrate and sulfate ions are 0.349 and 0.380 nm, respectively [28]. Nevertheless, it should be pointed out that these electrodialysis experiments were carried out for the solutions with a high content of organic solvent (30 wt % of MEA) that shall certainly influence the dissociation rate and ions mobility; thus, it can be expected that the observed specific HSS anions selectivity and transport rates can vary from the corresponded parameters obtained in the aqueous solutions with no presence of MEA. This can be confirmed by studies on the behavior of ion-exchange membranes in aqueous-organic media [29,30,31,32]. Unfortunately, the evaluation of effect of MEA presence on HSS anions transport was not part of this study, and it requires further investigation.

**Figure 6:**
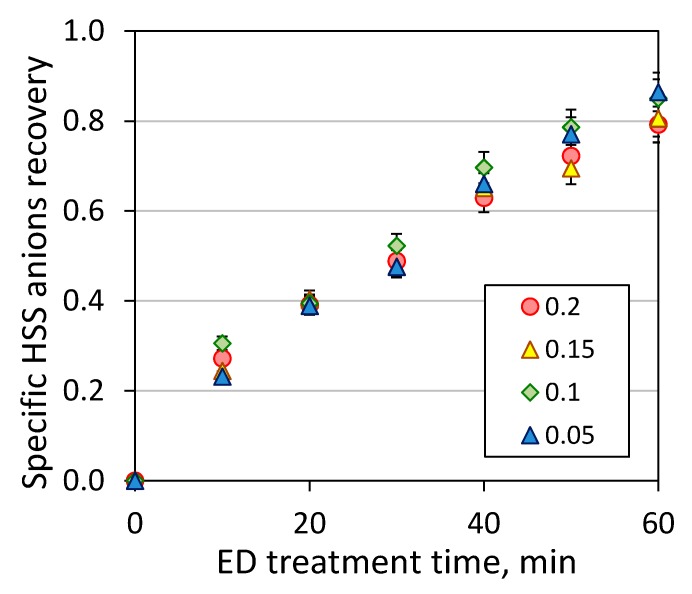
Kinetic of total HSS anions recovery during the second stage of ED reclaiming (ED II).

Based on the data presented on Figure 6, some general trends can be observed. Particularly, the transport of HSS anions in electrodialysis is governed by the anions charge and in a lesser extent by the acidity constants, and the efficiency of recovery of individual HSS anions are changed in the following order: nitrate > formate, acetate > sulfate, oxalate. Despite the difference in the concentrations (Table 1), formate and acetate anions possessed a very close recovery rate; the same observation was found for the pair of dibasic inorganic and organic acids—sulfate and oxalate anions, respectively. The presence of CO_2_ in the amine solution affects on the recovery of HSS anions from the feed solution, and the efficiency of ED reclaiming is dropped down with the increase of CO_2_ loading (see also Figure 4) because of an increase of charged species and their competiveness transport through the ion-exchanged membranes. This effect was less pronounced for strong, monobasic acid because the nitrate anions were completely removed from the feed within 30–40 min of ED treatment with or without the presence of carbon dioxide. More detailed study of each individual ion transport was out of scope of this work due to the complexity of multicomponent system and equilibrium presence of different products of interaction of carbon dioxide with aqueous monoethanolamine solution (carbamates, carbonates, bicarbonates, monoethanolammonium).

Upon 60 min of ED treatment (ED I), the concentrate streams contained a quite similar composition of HSS anions. Then, all concentrate streams loaded with carbon dioxide were treated with electrodialysis towards further concentration of HSS anions and reduction of waste stream. Figure 6 represents total HSS anions recovery from amine solutions as a function of time of ED reclaiming. It can be seen that all the amine solutions regardless the initial CO_2_ loading behaved quite similar unlikely to the first stage of electrodialysis. Such observation can be explained by very close values of electrical conductivity 17–23 mS/cm for all concentrate solutions generated after the first stage of ED treatment. For instance, the electrical conductivity of model MEA solutions used as a feed for ED I was varied in the wider range: model MEA solution with CO_2_ loading of 0.2 mole/mole 19 mS/cm, 0.15 mole/mole 15 mS/cm, 0.1 mole/mole 10 mS/cm, 0.05 mole/mole 6.7 mS/cm and 0 mole/mole 0.8 mS/cm. The typical content of HSS anions in the concentrate stream, which was later used as the feed solution for ED II, was as follows: formate ~44.5 mmeq/L, acetate ~8.5 mmeq/L, nitrate ~6.5 mmeq/L, sulfate ~12 mmeq/L, and oxalate ~16.5 mmeq/L.

### 3.2. Transport of MEA and CO_2_

**Figure 7:**
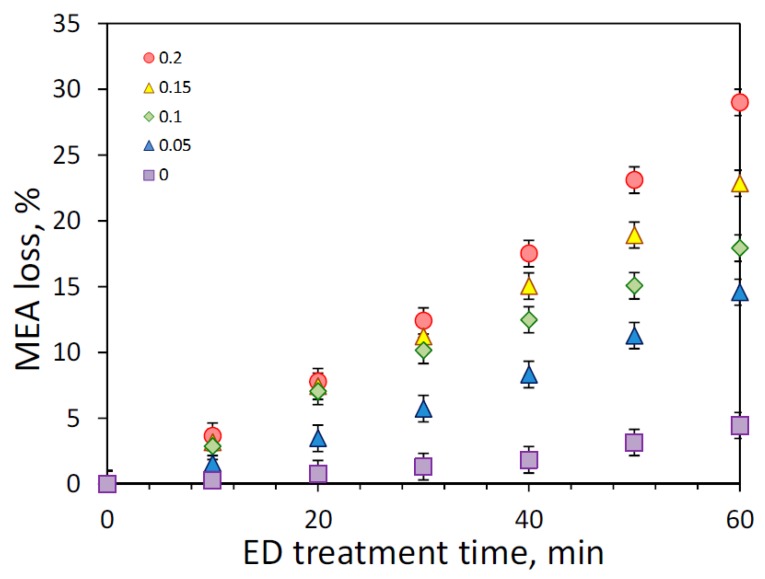
Monoethanolamine (MEA) loss at the first stage of ED reclaiming (ED I) of model MEA solution at the different solution CO_2_ loading.

**Figure 8:**
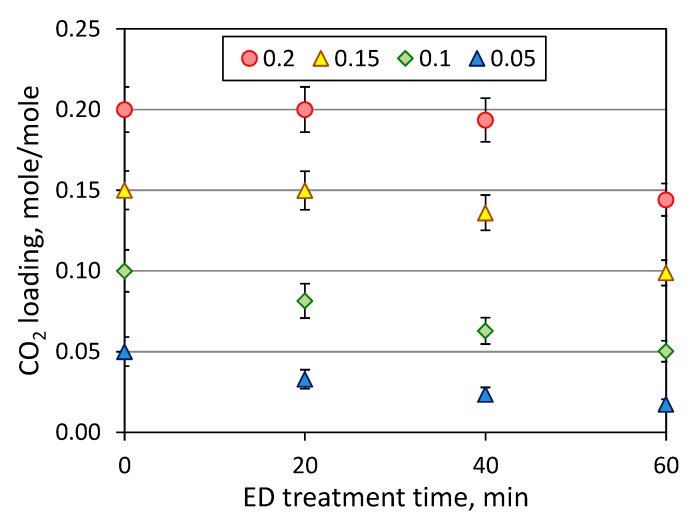
CO_2_ loading of MEA solutions at the first stage of ED reclaiming (ED I) of model MEA solution.

As discussed earlier, the presence of carbon dioxide might dramatically impact on the effectiveness of reclaiming process due to an increase of amine loss. Figure 7 shows the effect of initial CO_2_ loading on the MEA loss, which was determined as part of amine molecules migrated through the membrane from the feed to concentrate compartment during the electrodialysis. Since the major amine loss is attributed to the transfer of MEA molecules in the CO_2_-bound state (carbamate ions) or in the protonated form (alkanolammonium ion), the increase of CO_2_ loading led to greater MEA loss—from 4.4% (zero loading, 60 min) up to 29% (0.2 mole/mole, 60 min). In the case of absence of carbon dioxide, MEA molecules still transferred from the feed to the concentrate compartment as a counter ion (alkanolammonium) to HSS anions, and partially due to the electroosmotic transport through the anion exchange membrane as a part of the anion hydrated shells as noticed in [33]. Some insight can be given by considering the change of CO_2_ loading during ED (Figure 8). It can be seen that there is continuous decline of CO_2_ loading from the beginning of reclaiming of amine solutions with the initial carbon dioxide content of 0.05 and 0.1 mole (CO_2_)/mole (MEA); whereas, CO_2_ loading remained nearly unchanged during the first 20 min of ED treatment of amine solutions with higher CO_2_ content. These findings revealed a general tendency for faster depletion of carbon dioxide (presented mainly as carbonate and bicarbonate) over monoethanolamine (presented in different forms) in the feed solution during the electrodialysis.

Table 2 summarizes the molar flux of major components (MEA, CO_2_ and HSS anions), the relative MEA loss with the respect to removed HSS and CO_2_, and specific power consumption normalized by recovered HSS anions. There is a clear evidence that the appearance of carbon dioxide in amine solution resulted in the dramatic increase of MEA loss. For instance, the removal of 1 mole-equivalent of HSS anions from unloaded amine solution was associated with the transfer of 2 moles of MEA (see ED I in Table 2); meanwhile, the same ratio was changed by a factor of 11 for amine with CO_2_ loading of 0.2 mole/mole—from 2.0 up to 22.1 mole (MEA)/mole-equivalent (HSS). As expected, appearance of greater number of charged species in the common solution shall lead to increase of power consumption required to achieve the same recovery rate of target components. Therefore, the complete regeneration of amine solvent from 0.2 down to 0 mole/mole, as an ideal case scenario, might reduce the specific energy consumption from 140 down 37 kJ per 1 g of removed HSS anions. Thereby, the decrease of carbon dioxide content in MEA provides not only the reduction in MEA loss, but also a certain intensification of electrodialysis process.

The main goal of the second ED treatment was the reduction of volume of waste stream came from the first stage by a factor of two, and consequently reduce MEA loss: 28→14% (initial CO_2_ loading of 0.2 mole/mole), 22→12% (0.15 mole/mole), 20→10% (0.1 mole/mole), and 14→7% (0.05 mole/mole). The amine loss associated with the further recovery of HSS from different concentrated solutions at the second stage of ED reclaiming was varied in the closer range—from 5.4 up to 9.9 mole (MEA)/mole-equivalent (HSS) as can be noticed from the data for EDII in Table 2. In all cases, the specific electric energy consumption for ED II was within the range of 76–80 kJ/g (HSS). Besides, the diluate stream can be recycled back to the first stage as the feed due to quite close concentration of HSS anions.

### 3.3. Comparison of Power Consumption: ED Reclaiming vs Addition CO_2_ Stripper

As shown above, the additional CO_2_ stripping from the lean amine solution looks very attractive towards improvement of electrodialysis reclaiming of HSS anions and reduction of MEA loss. However, the drawback of this concept is the additional expenditures required for regeneration of amine solvent at higher temperature and/or operation of additional stripping column. In this work, we have made an attempt to estimate the power consumption required for deeper regeneration of initial lean MEA solution with CO_2_ loading of 0.2 mole/mole. In this work a number of simplifications were presumed for this calculation: (1) only MEA solution fed to the electrodialysis purification was subjected of deeper regeneration; (2) the design and size of stripping column was fixed for all MEA solutions considered, and only the power consumption for the desorption process was determined; (3) HSS content was fixed as 48 meq/L for all different feed streams for ED process; (4) similarly to work [25] the rate of HSS extraction was taken equal to 7 eq/h at the 100 m^3^/h flow rate of the MEA-absorbent withdrawn for the separation. The calculations were made for system consist of stripping column and the ED reclaimer with the capacity of 100 m^3^/h of lean MEA.

At the first stage, energy consumption was estimated for HSS removal in a large-scale two-stage ED reclaimer. The main parameters of the electrodialyzer are the area of the active membrane, specific energy consumption and flowrate of the extracted components. The specific characteristics of EDI and EDII stages were taken from Table 2. For each CO_2_-loading of lean MEA solution, the ED unit was design to maintain the following flowrate of HSS anions transport from the feed to the concentrate compartment at the constant level: 7 eq/h for EDI and 9.6 eq/h for EDII. Such rate of HSS removal allows maintaining their level in the absorption system below the critical value, after which the avalanche-like increase of MEA degradation products takes place as reported earlier [34]. The main parameters of the two-stage ED reclaimer with the capacity of 100 m^3^/h of lean MEA is presented in electrodialyzers of the EDI and EDII stages for each studied CO_2_ load are listed in Table 3. Clearly, HSS removal would be conducted with the most efficiency from the solution with zero CO_2_ loading; however, it is impossible to fully strip the absorbent solution from CO_2_ in real conditions. Nevertheless, the decrease of the CO_2_ loading from 0.2 to 0.15 mole (CO_2_)/mole (MEA) leads to the reduction of the required active electrodialyzers area of the two-stage system by 28%, decline of the energy consumption by 45% and MEA flux to the concentrate by 60%.

**Figure 9:**
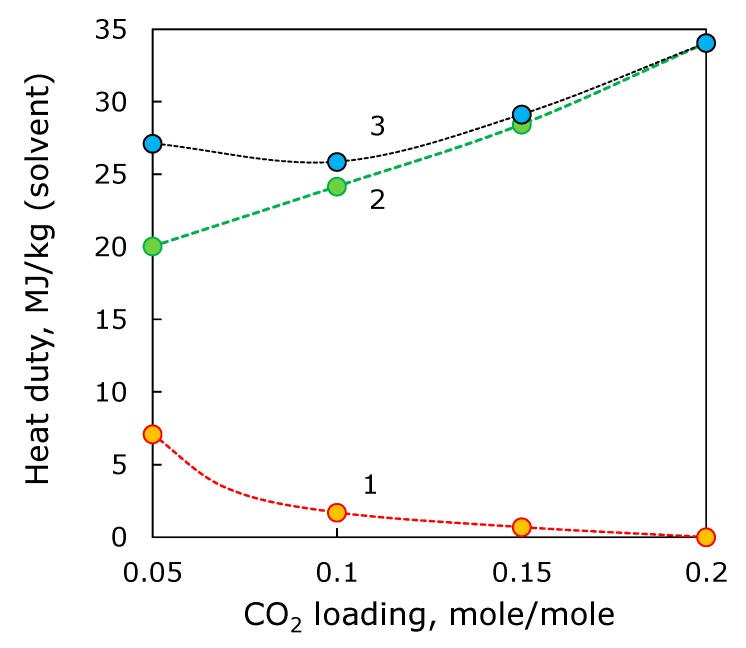
The heat duty of reduction of CO_2_ loading from 0.2 mole/mole down to require level (1), ED reclaimer (2) and overall energy consumption (3) for combination of two units.

The stripping column that enables to achieve CO_2_ loading lower than 0.2 mole/mole was simulated in Aspen Plus^®^. As mentioned above, the mass-transfer surface area was deliberately set higher than necessary to provide the equal conditions for all cases. To compare the heat and electricity energy consumption required for stripping column and ED reclaimer, the coefficient of 3.46 was used. The ED process, the energy consumption was directly calculated for 1 kg of 30 wt % MEA solution in water. Figure 9 shows the specific heat energy consumption required for (i) reduction of CO_2_ loading in MEA stream of 100 m^3^/h from 0.2 mole/mole down to desired level, (ii) removal of HSS anions from MEA stream after the stripping, and (iii) performance of hybrid process based on stripping and ED reclaiming. As can be seen, the electrodialysis might provide a major contribution to the heat duty comparing with the stripping column. Based on rough estimation of the heat duties, it seems that the CO_2_ loading of 0.1 mole/mole can be worth of further investigation.

## 4. Conclusions

In this work, it was shown that the decrease of carbon dioxide content from 0.2 down to 0 mole (CO_2_)/mole (MEA) resulted in a substantial reduction of the specific energy consumption from 140 down 37 kJ per 1 g of recovered HSS anions. This can be explained by the drop of the total concentration of ions formed by the interaction of MEA solution with carbon dioxide. The removal of 1 mole-equivalent of HSS anions from unloaded amine solution was associated with the transfer of 2 moles of MEA; meanwhile, the same ratio was changed by a factor of 11 for amine with CO_2_ loading of 0.2 mole/mole—from 2.0 up to 22.1 mole (MEA)/mole-equivalent (HSS). It was shown that the transport of HSS anions in electrodialysis is governed by the anions charge and in a lesser extent by the acidity constants, and the efficiency of recovery of individual HSS anions are changed in the following order: nitrate > formate, acetate > sulfate, oxalate.

The change of CO_2_ loading is associated with additional power consumption towards further solvent regeneration in the column. Based on the preliminary estimations of power consumption required for additional CO_2_ stripping with the respect to the power consumption of ED stage, it seems that CO_2_ loading of 0.1 mole/mole provides an optimum for the power input at 25.9 MJ/kg(solvent).

## Figures and Tables

**Table 1:** The content of HSS components (anions) in the model absorbent solution.

Anion HSS	Concentration (mg/L)
Formate (HCOO^−^)	1065
Acetate (CH3COO^−^)	225
Oxalate (C_2_O_4_^2−^)	392
Nitrate (NO_3_^−^)	234
Sulfate (SO_4_^2−^)	390

**Table 2:** Some characteristics of the first ED stage (total initial HSS content—48 meq/L) and the second ED stage (total initial HSS content—89 meq/L).

ED Variant	Initial CO_2_ Loading, mole (CO_2_)/mole (MEA)	Molar Flux, mole/(m^2^∙h)			MEA Loss, mole/mole СО_2_ (HSS)		Specific Energy Consumption, kJ/g HSS
		MEA	СО_2_	HSS mole-eq	MEA/СО_2_	MEA/HSS	
ED I	0.20	8.50	2.79	0.384	3.1	22.1	140
	0.15	7.38	2.5	0.408	3.0	18.1	113
	0.10	6.67	2.4	0.451	2.8	14.8	92
	0.05	3.64	1.5	0.524	2.4	7.0	69
	0	1.73	-	0.858	-	2.0	37
ED II	0.20	8.97	4.18	0,904	2.2	9.9	80
	0.15	8.50	3.84	0.999	2.2	8.5	79
	0.10	7.82	3.26	1.057	2.4	7.4	76
	0.05	6.38	2.56	1.178	2.5	5.4	76

**Table 3:** The main parameters of two-stage of electrodialysis reclaimer with the capacity of 100 m^3^/h of lean MEA.

α	ED Stage	Active Membrane Area (m^2^)	Specific Power Consumption (MJ/kg HSS)	Flow Rate to the Concentrate (mole/h)		
				MEA (MEA loss)	СО_2_	HSS (mole-eq/h)
0.2	EDI	25	150	215	70	9.6
	EDII	8	90	70	33	7.0
	EDI + EDII	33	240	70	33	7.0
0.15	EDI	24	118	174	59	9.6
	EDII	7	90	60	27	7.0
	EDI + EDII	31	207	60	27	7.0
0.1	EDI	21	95	143	51	9.6
	EDII	7	86	52	22	7.0
	EDI + EDII	28	181	52	22	7.0
0.05	EDI	18	72	67	28	9.6
	EDII	6	85	38	15	7.0
	EDI + EDII	24	157	38	15	7.0
0	-	8	38	14	-	7.0
